# Phenotypic and Genetic Determination of Biofilm Formation in Heat Resistant *Escherichia coli* Possessing the Locus of Heat Resistance

**DOI:** 10.3390/microorganisms9020403

**Published:** 2021-02-15

**Authors:** Angela Ma, Norman Neumann, Linda Chui

**Affiliations:** 1 Department of Laboratory Medicine and Pathology, University of Alberta, Edmonton, AB T6G 2R3, Canada; ama6@ualberta.ca; 2 School of Public Health, University of Alberta, Edmonton, AB T6G 2R3, Canada; nfneuman@ualberta.ca; 3 Alberta Precision Laboratories—Provincial Laboratory for Public Health, Edmonton, AB T6G 2J2, Canada

**Keywords:** *Escherichia coli*, biofilm, locus of heat resistance, multi-stress tolerance, food processing

## Abstract

Despite the effectiveness of thermal inactivation processes, *Escherichia* *coli* biofilms continue to be a persistent source of contamination in food processing environments. *E. coli* strains possessing the locus of heat resistance are a novel food safety threat and raises the question of whether these strains can also form biofilms. The objectives of this study were to determine biofilm formation in heat resistant *E. coli* isolates from clinical and environmental origins using an in-house, two-component apparatus and to characterize biofilm formation-associated genes in the isolates using whole genome sequencing. Optimal conditions for biofilm formation in each of the heat resistant isolates were determined by manipulating inoculum size, nutrient concentration, and temperature conditions. Biofilm formation in the heat resistant isolates was detected at temperatures of 24 °C and 37 °C but not at 4 °C. Furthermore, biofilm formation was observed in all environmental isolates but only one clinical isolate despite shared profiles in biofilm formation-associated genes encoded by the isolates from both sources. The circulation of heat resistant *E. coli* isolates with multi-stress tolerance capabilities in environments related to food processing signify that such strains may be a serious food safety and public health risk.

## 1. Introduction

*Escherichia coli* is ubiquitous as normal flora in humans and animals [1]. However, acquisition of virulence factors allows some *E. coli* strains to become pathogenic and consequentially, become etiological agents for a multitude of human infections. Of the pathogenic *E. coli*, pathotypes can be classified into two categories, intestinal and extraintestinal, based on virulence factors the strains possess and their corresponding clinical presentations in humans [2]. Intestinal *E. coli* pathotypes include enteroaggregative *E. coli* (EAEC), enteropathogenic *E. coli* (EPEC), enterotoxigenic *E. coli* (ETEC), enteroinvasive *E. coli* (EIEC), enterohemorrhagic *E. coli* (EHEC), and diffuse-adhering *E. coli* (DAEC) that typically present as diarrheal disease in human infection. Urinary tract infections, meningitis, and sepsis are commonly attributed to infection by extraintestinal *E. coli* pathotypes including uropathogenic *E. coli* (UPEC), neonatal meningitis-associated *E. coli* (NMEC), and sepsis-associated *E. coli* (SEPEC), respectively.

Biofilms are microbial aggregates living as a community through secretion of an extracellular polymeric matrix composed of exopolysaccharides, proteins, and DNA [3]. Such communities can persist on abiotic and biotic surfaces for extended periods of time due to their high resistance to disinfectants and antimicrobials. *E. coli* biofilms are responsible for a plethora of nosocomial, device-related infections and are a persistent source of contamination in the environment [4,5]. The ability of *E. coli* biofilms to withstand harsh conditions is achieved by quorum sensing, the chemical signalling pathway in which bacterial cells communicate with each other through secretion of autoinducer substances to mediate biofilm formation and maturation, and secretion of virulence factors [3]. EAEC and UPEC pathotypes have been thoroughly investigated for their biofilm forming potential [6]; however, research in biofilm formation in other pathotypes, including EHEC requires further investigation. EHEC is a zoonotic agent of foodborne illness and cattle can serve as a natural reservoir for the pathotype [7]. Routes of EHEC transmission include consumption of contaminated food or water, direct contact with animals, and person-to-person spread [8]. In conditions of poor sanitation in food processing, EHEC biofilms on equipment and surfaces can be a source of persistent contamination on carcasses and food products [9]. Processing plants routinely incorporate high temperature disinfectant solutions of 50 °C into their sanitation procedures to remove visible grime and also eliminate bacterial biofilms [10,11]. However, the circulation of EHEC strains possessing the locus of heat resistance (LHR), which confers exceptional thermotolerance to temperatures of 60 °C and above [12], may be problematic in food processing environments. Biofilm formation in both EHEC and heat resistant *E. coli* strains has not been well explored, but the presence of the organism may serve as a substantial threat to food safety [8,13]. Reports of heat resistant, clinical isolates have shown resistance to heat and osmotic stress at temperatures of 60 °C and 71 °C [14,15]. These temperatures are comparable to those used in the food processing industry’s heat inactivation procedures and reflective of temperatures recommended for safe consumer cooking.

The objectives of this study were to comparatively detect biofilm formation in clinical and environmental *E. coli* isolates possessing the locus of heat resistance using an in-house, two-component apparatus and characterize their genetic profiles in respect to biofilm formation-associated genes.

## 2. Materials and Methods

### 2.1. Bacterial Isolates and Growth Conditions

Heat resistant *E. coli* isolates included originated from both clinical and environmental sources; three clinical isolates previously identified as 111, 128, and 8354 were from human cases of acute gastroenteritis submitted to Alberta Precision Laboratories—Provincial Laboratory for Public Health (ProvLab) [15]. Environmental isolate AW1.7 originated from a local cattle slaughter plant [16] and isolates 53 and 63 were obtained from a municipal wastewater treatment plant [17]. The six heat-resistant isolates were of unknown pathotypes. All of these isolates were recovered from frozen skim milk stocks and streaked onto sheep blood agar plates (BAP) (Dalynn Biologicals, Calgary, AB, Canada) and incubated for 24 h at 37 °C for use in subsequent experiments.

### 2.2. Detection of Biofilm Formation by Crystal Violet Staining

**Figure 1:**
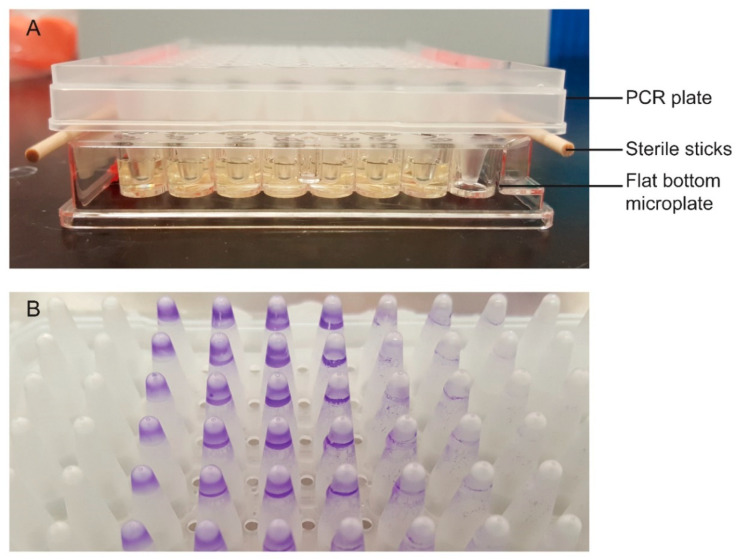
Two-component apparatus for detecting biofilm formation (**A**). The pegs of the PCR plate are submerged into wells of the flat bottom microplate containing bacterial cells. The PCR plate rests on top of 2 sterile sticks to prevent the pegs from direct contact with the bottom of the microplate. Biofilms form on the pegs that are subsequently stained with 1% crystal violet (**B**).

Biofilm formation for the six heat-resistant *E. coli* isolates was detected using an in-house, two-component apparatus (Figure 1A). A single colony from each BAP culture was inoculated into 10 mL of Luria Bertani (LB) broth (Becton Dickinson, Mississauga, ON, Canada) followed by incubation at 37 °C for 24 h with agitation. Stationary phase cultures of the isolates were adjusted to an optical density (OD) at 600 nm of 0.5 (Microscan Turbidity Meter, Siemens, Oakville, ON, Canada) with phosphate buffered saline (pH 7.0) from which 1 mL aliquots were washed by centrifugation at 20,000× *g* for 2 min and re-suspended with 1 mL of saline. For each isolate that biofilm formation was to be determined, the wells of a 96 well flat bottom clear polystyrene microplate (Corning; Millipore Sigma, Milwaukee, WI, USA) were filled with 140 µL of LB broth in triplicate. To these wells, 10 µL of the aliquots of bacterial cells were added; 10 µL of saline was added to 140 µL of LB broth to serve as a blank. Sterile sticks (Puritan 6” wooden applicator stick; Puritan Medical Products, Guilford, ME, USA) were taped onto the longitudinal sides of the microplate to ensure that the pegs of the 96 well PCR plate (MicroAmp; Thermo Fisher Scientific, Waltham, MA, USA) that was laid on top of the microplate would not come in direct contact with it. The pegs of the PCR plate were submerged into the wells of the flat bottom microplate inoculated with bacterial culture or saline and the top and bottom components of the apparatus were sealed together with additional tape. The apparatus was stored in an air-tight plastic container lined with damp paper towels to prevent evaporation over a 24 h incubation period at 4 °C. Unlike conventional biofilm assays that typically assess static biofilm formation from the wells of the flat bottom microplate, biofilms formed by motile bacterial cells were detected from the pegs of the PCR plate. Following incubation, the PCR plate was disassembled from the apparatus and washed in 200 mL of Milli-Q water for 30 s with light agitation by hand (4x). The washed PCR plate was then turned with the pegs facing upwards to remove excess Milli-Q water and dried for 10 min. To a 96 well round bottom clear polystyrene microplate (Corning; Millipore Sigma), 200 µL of crystal violet (Millipore Sigma) diluted to 1% with Milli-Q water was added to each well corresponding with the wells of the flat bottom microplate. The PCR plate was laid on top of the round bottom microplate so that the pegs were submerged in the crystal violet solution for 30 min at 24 °C. Following staining, the PCR plate was again washed in 200 mL of Milli-Q water with light agitation for 30 s (4x) and dried for 10 min (Figure 1B). Lastly, a second round bottom microplate was prepared with 150 µL of 95% ethanol per well for de-staining of the pegs. The PCR plate was immersed in the round bottom microplate for 30 min at 4 °C during which any crystal violet adhering to the pegs dissolved into the ethanol. Absorbance of crystal violet was measured at 595 nm using a SpectraMax 190 Microplate Reader (Molecular Devices LLC, San Jose, CA, USA) and SoftMax Pro software. Screening for biofilm formation in LB broth was repeated for all isolates at incubation temperatures of 24 °C and 37 °C.

### 2.3. Determination of Optimal Biofilm Formation Conditions

After initial screening for biofilm formation in LB broth, experimentation to determine the optimal conditions such as inoculum size, nutrient concentration, and temperature conditions for biofilms were performed for each isolate using the two-component apparatus. Modifications for this experimental procedure required serial dilutions of un-inoculated, sterilized LB broth with Milli-Q water to obtain concentrations from 100% to 10% in decreasing intervals of 10%. After adjustment to an OD of 0.5 at 600 nm with phosphate buffered saline (pH 7.0), stationary phase cultures were again washed by centrifugation and then serially diluted with saline to obtain cell concentrations ranging from 8 log CFU/mL to 1 CFU/mL over 5 dilutions. The flat bottom microplate was subsequently inoculated with 140 µL of LB broth dilutions and 10 µL of inoculum dilutions of cells according to a plate map; lastly a PCR plate was overlaid on top of the microplate as described above. Incubation conditions, staining and de-staining procedures, and measurement of absorbance were performed as previously described.

### 2.4. Genomic DNA Isolation, Whole Genome Sequencing, and Analysis for Biofilm-Associated Genes

Genomic DNA of heat resistant *E. coli* isolates was extracted from overnight cultures grown on BAP using the MagaZorb DNA mini-prep kit (Promega Corporation, Madison, WI, USA). The Qubit 4 Fluorometer (Invitrogen, Burlington, ON, Canada) was used to determine the quality and quantity of DNA. Sequencing libraries were prepared using the Nextera XT kit (Illumina Inc., San Diego, CA, USA). Whole genome sequencing was performed using the Illumina MiSeq platform (Illumina Inc.) according to the manufacturer’s instructions. MiSeq sequencing runs were performed with paired-end 250-nucleotide reads. Trimmomatic Version 0.38 [18] was used to trim the low-quality reads of each genome with the following parameters: SLIDINGWINDOW = 4:15, LEADING = 3, TRAILING = 3 MINLEN = 36. De novo assembly was performed using SPAdes Version 3.9.1 [19] with ‘--careful’ and ‘-k 21,33,55,77’ options. All genomic sequences of the 6 isolates were deposited to the NCBI genome database under BioProject PRJNA694975. Presence or absence of biofilm formation-associated genes (Table 1) in each of the isolates was identified using the NCBI BLAST server.

### 2.5. Motility Status

Presence or absence of *fliC* (gene ID: 949101) as a genetic marker for motility was determined for all isolates using the NCBI BLAST server against their respective genomes. Phenotypic expression for flagellin was confirmed for all isolates with two motility tests: triphenyltetrazolium chloride (TTC) medium (Dalynn Biologicals, Calgary, AB, Canada) and sulfide, indole, motility (SIM) medium (Dalynn Biologicals). Isolated colonies from overnight cultures were inoculated from the BAP into the motility test media by stabbing the center to a depth of approximately 1.5 inches. The test media were incubated overnight at 37 °C. Extension from the stab line for growth as visualized by turbidity or cloudiness, and diffusion of formazan in the case of TTC medium were considered positive indicators for motility. *E. coli* ATCC 25922 and *Klebsiella pneumoniae* ATCC 700603 were used as a positive and negative control, respectively.

### 2.6. Statistical Analysis

The means of at least three independent experiments were determined to optimize the experimental procedure of the two-component apparatus for each isolate at all incubation temperatures. Biofilm formation between environmental and clinical isolates at each incubation temperature without modifications to inoculum size and nutrient concentration was compared using a Two-Sample *t*-Test with OriginPro 2016 (OriginLab, Northampton, MA, USA). A 95% significance (*p* = 0.05) was used. The means of at least 3 independent experiments were determined for all serial dilutions of inoculum and LB broth for each isolate when determining optimal biofilm formation conditions and the data was analyzed by linear regression with R 4.0.3 [34].

## 3. Results

### 3.1. Virulence Characteristics and Motility of E. coli Isolates

**Figure 2:**
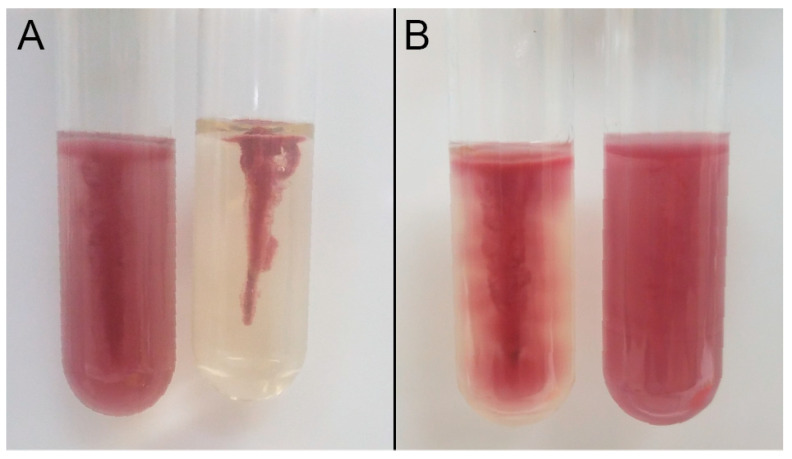
Triphenyltetrazolium Chloride (TTC) motility test media of (**A**) positive control *Escherichia coli* ATCC 25922 (left) and negative control *Klebsiella pneumoniae* ATCC 700603 (right) and (**B**) heat resistant isolates AW1.7 (left) and 128 (right). Results from TTC media of isolates 53, 63, and 111 were identical to isolate AW1.7 and isolate 8354 was identical to isolate 128.

Characterization of the clinical isolates for key virulence factors of the EHEC pathotype including *stx*_1_, *stx*_2_, and *eae*, was previously determined by Chui et al. [35]. Isolate 111 (serotype ONT:H25) was positive for *stx*_1_ and *eae* and negative for *stx*_2_, isolate 128 (serotype O11:H25) was positive for *stx*_1_ and negative for *stx*_2_ and *eae*, and isolate 8354 (serotype O157:H7) was negative for *stx*_1_, *stx*_2_, and *eae*. Despite clinical isolate 8354 lacking the genetic predictors of EHEC, it was the sole etiologic agent identified from a case of acute gastroenteritis. Expectedly, environmental isolates AW1.7 (serotype O128:H12), 53 (serotype O11:H25), and 63 (serotype O11:H25) were negative for EHEC virulence factors including *stx*_1_, *stx*_2_, and *eae*. All six heat resistant isolates possessed the *fliC* gene encoding for flagellin and were positive for phenotypic motility as confirmed by TTC and SIM media. Interestingly, motility was not the same for all isolates when observed by TTC medium (Figure 2). Isolates AW1.7, 53, 63, and 111 showed less diffusion of formazan compared to isolates 128 and 8354 as observed by the spread of pigment from the stab line. All isolates showed a diffuse zone of growth from the stab line in SIM medium.

### 3.2. Optimization of the Two-Component Apparatus for E. coli

**Figure 3:**
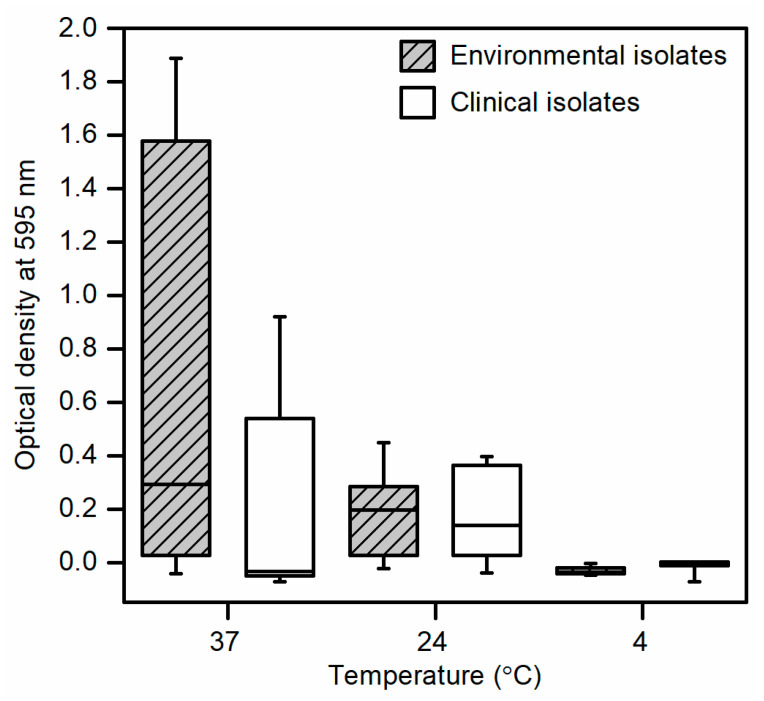
Biofilm formation of environmental and clinical *Escherichia coli* isolates in 100% LB broth and 8 log CFU/mL inoculum size incubated at temperatures of 37 °C, 24 °C, and 4 °C for optimization of the two-component apparatus. Data presented as means ± standard deviations of triplicate experiments.

Initial screening and optimization of the two-component apparatus using bacterial cultures standardized to 8 log CFU/mL revealed biofilm formation in all environmental isolates and clinical isolate 111 at temperatures of 24 °C and 37 °C but not at 4 °C (Figure 3). No biofilm formation was detected from clinical isolates 128 and 8354 at all incubation temperatures. At an incubation temperature of 37 °C, environmental isolates showed increased biofilm formation compared to clinical isolates whereas at 24 °C, biofilm formation in both groups were similar. No statistical differences in biofilm formation were observed between environmental and clinical isolates at all incubation temperatures.

### 3.3. Determination of Optimal Biofilm Formation Conditions

**Figure 4:**
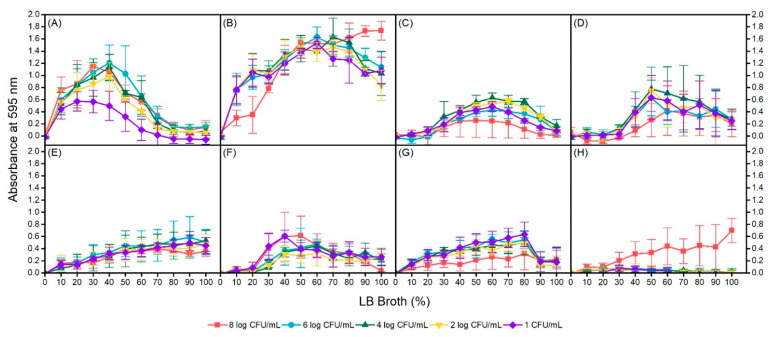
Biofilm formation in heat resistant *Escherichia coli* environmental isolates AW1.7 at 24 °C (**A**) and 37 °C (**B**); 53 at 24 °C (**C**) and 37 °C (**D**); 63 at 24 °C (**E**) and 37 °C (**F**); and clinical isolate 111 at 24 °C (**G**) and 37 °C (**H**) under conditions of inoculum size and nutrient concentration manipulation. Data presented as means ± standard deviations of triplicate experiments.

To determine the optimal biofilm formation conditions for each isolate, manipulation of inoculum size and nutrient concentration was incorporated through serial dilutions of each variable. Again, similar observations of no biofilm formation were obtained at 4 °C for all isolates regardless of inoculum size and nutrient concentration. For the 4 isolates that were capable of forming biofilms at 24 °C and 37 °C, conditions yielding maximum biofilm formation were not identical within isolates at different incubation temperatures or between isolates at the same temperatures (Figure 4). Among the isolates, environmental isolate AW1.7 formed the most biofilm at 24 °C and 37 °C temperatures as measured by absorbance of crystal violet at 595 nm. In addition, biofilm formation of AW1.7 was evidently different between the two temperatures, with maximum formation in 40% LB broth and 6 log CFU/mL inoculum, and 100% LB broth and 8 log CFU/mL inoculum at 24 °C and 37 °C, respectively. Environmental isolate 53 was capable of forming biofilms at 24 °C in concentrations of 30% to 90% LB broth at all cell concentrations with the exception of 8 log CFU/mL and maximum formation was observed at 60% LB broth and 4 log CFU/mL. Biofilm formation in isolate 53 was interestingly quite different at 37 °C, with maximum formation at 50% LB broth for most cell concentrations. Furthermore, biofilm formation at this temperature was undetectable or weak until a nutrient concentration of 40% LB broth was reached. Isolate 63, also of environmental origin, was capable of forming biofilms at both 24 °C and 37 °C. At 24 °C, biofilm formation was linear, with maximum formation observed at 90% and 100% LB broth at all cell concentrations. Similar to isolate 53, at 37 °C, isolate 63 was capable of forming biofilms only in specific conditions; LB broth concentrations of 0% to 20% did not support biofilm formation at any inoculum sizes. Optimal conditions for maximum biofilm formation in isolate 63 at 37 °C were observed at 40% LB broth and 1 log CFU/mL, and 50% LB broth and 8 log CFU/mL. Of the 3 clinical, heat resistant isolates, only isolate 111 was a biofilm former. Isolate 111 formed biofilms at all inoculum sizes at 24 °C incubation but maximum formation was observed only up to 80% LB broth. In addition, biofilm formation was substantially lower at an inoculum size of 8 log CFU/mL regardless of LB broth concentration compared to the other dilutions. Interestingly, at 37 °C, only an inoculum of 8 log CFU/mL of isolate 111 had biofilm formation. All other cell dilutions of isolate 111 were unable to form any biofilm across all LB broth concentrations. Upon regression analysis, statistically significant interactions between temperature and nutrient concentration were observed for isolates AW1.7, 53, and 63; no interaction was detected for clinical isolate 111 (Table 2). In isolate 63, increasing the bacterial inoculum was not significantly associated with absorbance of crystal violet (*p* = 0.791, 95% CI: (−0.006, 0.007)). However, in isolates AW1.7 and 111, an increasing bacterial inoculum was associated with an increase in mean absorbance whereas in isolate 53, a decrease in mean absorbance was detected.

### 3.4. Genetic Analysis of Biofilm Formation-Associated Genes

Genome sequence data was used to determine the presence or absence of known biofilm formation-associated genes for the six heat-resistant isolates (Table 3). All isolates possessed the *csgBAC* and *csgDEFG* operons but lacked the *mrkABCDF* operon, which encodes for curli and type 3 fimbriae, respectively. Genetic profiles for biofilm formation-associated genes in environmental isolates 53 and 63, both of which originated from wastewater, and clinical isolate 111 were identical. However, optimal conditions for biofilm formation in all 3 of these isolates were noticeably different from each other. Despite isolates AW1.7 and 8354 both possessing the same genetic profile for biofilm formation-associated genes, only the former was capable of forming biofilm.

## 4. Discussion

Biofilm formation using the in-house, two-component apparatus was investigated in six heat-resistant *E. coli* isolates. Heat resistant *E. coli* isolates AW1.7, 53, and 63 obtained from the environment served as a comparison of a different isolation source from the three clinical isolates. Furthermore, wastewater isolates 53 and 63 were previously determined to be biofilm formers in tryptic soy broth at 35 °C [17,36]. All 3 environmental isolates were capable of forming biofilms whereas biofilm formation was detected only in clinical isolate 111. Furthermore, isolate AW1.7 was most proficient at biofilm formation at 37 °C compared to isolates 53 and 63. This difference may be attributed to their respective sources of isolation. External stressors significantly influence an organism’s potential for biofilm formation [37,38]. Isolate AW1.7 originated from a local cattle slaughter plant and may be highly adapted to temperatures of 37 °C and above that reflect the gastrointestinal tract of ruminants [39] and thermal inactivation processes used in beef processing. On the other hand, isolates 53 and 63 were obtained from municipal wastewater, which ranges in temperatures between 4 °C and 20 °C [17]. Although these isolates were unable to form biofilms at 4 °C, biofilm formation was still detected 24 °C and 37 °C. Furthermore, it has been documented that isolates 53 and 63 possess high levels of resistance to chlorine and peroxide in addition to heat resistance [17,36], adaptations reflective of their environmental origin.

Conventional biofilm procedures measure total static biofilm production on the bottom of a microplate well following incubation by staining with crystal violet and measuring the absorbance. However, a disadvantage to this method is that matrix components and dead cells might settle at the bottom of the well and uptake the stain, which may lead to a potential over-estimation of biofilm formation [40]. The in-house, two-component apparatus detects biofilm formation on the underside of the PCR plate from motile cells, eliminating any over-estimation as a result of inadequate washing of the microplate wells in the conventional method. Various factors such as inoculum size and concentration of nutrient media can be manipulated and added to the same microplate for evaluation of their effect on biofilm formation. Furthermore, the two-component apparatus utilizes PCR plates made of polypropylene but the material of the pegs on which the biofilms form can easily be substituted or coated with an array of different materials or substances to facilitate biofilm formation in fastidious organisms or to assess susceptibility to antimicrobials and disinfectants. Resemblances are shared between the two-component apparatus discussed in this study and the commercially available MBEC Assay (Innovotech Inc., Edmonton, AB, Canada), formerly the Calgary Biofilm Device [41]. With limited research funding available, the in-house, two-component apparatus can be used as a substitute to test for biofilm formation in a variety of bacteria.

Current pathogen intervention steps for cattle carcasses in food processing plants include hot water, steam pasteurization, and antimicrobial solutions applied at high temperatures ranging from 60 °C to 95 °C [10,11]. Sanitation of knives and blades during slaughter also regularly occur at 82 °C [42]. During such processes, buildup of mud and feces from contaminated hides on processing equipment may occur. Poor plant sanitation can allow for biofilm formation on these surfaces and become a source of contamination [9]. Many processing plants incorporate high temperatures into their cleaning procedures but limited research has been conducted thus far on whether heat resistance contributes to increased survival of *E. coli* in biofilms [43]. Furthermore, previous studies on the effect of environmental stressors on EHEC found it unlikely that strains could possess virulence genes for human infection and phenotypes for evading pathogen intervention measures in the food processing industry [44]. However, clinical isolate 111 that possesses Shiga toxin, the locus of enterocyte effacement (LEE), the LHR, and the biofilm forming phenotype was characterized in this study, indicating that pathogenic *E. coli* strains with heightened survival traits are in circulation and may represent a significant food safety threat. Future studies on assessing the survival of biofilms formed by heat resistant *E. coli* after exposure to high temperatures reflective of those used in food processing may provide additional insight on its persistence in food processing plants.

The genes involved in biofilm formation and those related to quorum sensing belong to a highly complex field of study that is ever expanding. This study presents genetic analysis of genes associated with biofilm formation in *E. coli*, particularly Shiga toxin-producing *E. coli* (STEC), which has been poorly investigated thus far. Active motility through flagellin has previously been described as a requirement for biofilm formation in *E. coli* [45,46] in order to overcome the repulsive electrostatic and hydrodynamic forces in a liquid environment [47]. However, non-motile *E. coli* K-12 and EAEC strains have been reported to form biofilms by overexpression of surface adhesins to compensate for the lack of force-generating movements provided by flagellin [48,49]. All heat resistant isolates were motile, fulfilling the first prerequisite for biofilm formation. The LEE is a pathogenicity island that defines the EHEC pathotype and plays a vital role in the development of attaching and effacing lesions, and adherence to intestinal epithelial cells during pathogenesis [50]. Despite the fact the three clinical, heat-resistant isolates originated from cases of acute gastroenteritis that would likely be indicative of EHEC infection, only isolate 111 was positive for the LEE, as determined by detection of the *eae* gene. The adhesin intimin is encoded by the *eae* gene, one of 41 open reading frames in the LEE [51]. Regulation of the LEE is mediated by quorum sensing, specifically by the *luxR* homolog *sdiA* [3,9], and the SdiA protein has been proposed to be involved in biofilm formation in *E. coli*. It is speculated that regulation of the LEE through SdiA may also be reflective of the isolate’s ability to form biofilms and is supported by the findings in this study. Isolate 111 was the only clinical isolate that formed biofilms and also possessed the LEE.

Curli and cellulose are two commonly studied markers of biofilm formation as they are major components of the biofilm matrix [22,24]. Of the heat resistant *E. coli* isolates sequenced, the *csgBAC* and *csgDEFG* operons that encode for curli were present and with the exception of clinical isolate 128; the *bcsA* and *bcsB* genes, which encode for cellulose, were identified in all remaining isolates. However, biofilm formation was only detected in isolates AW1.7, 53, 63, and 111, indicating that genetic and/or phenotypic characterization of curli and cellulose does not correspond with biofilm forming potential and should not be used as the sole methods to investigate biofilm formation. All biofilm forming isolates possess the *pgaC* gene but lack the *papC* and *Agn43* genes. *pgaC* encodes for the synthesis of PGA polymer, which plays a role in biofilm adhesion [25]. However, PGA is not the only adhesion factor in biofilm formation as LPS, curli, fimbriae, and pili are also involved in this process. Understandably, all clinical isolates were negative for the *papC* and *Agn43* genes that encode for adhesion factors P pili and autoaggregation factor antigen 43, respectively. P pili are commonly found in UPEC strains as they are critical adhesion factors for the pathogenesis of ascending urinary tract infections [52]. Autoaggregation factor antigen 43 has been reported in high prevalence in UPEC, SEPEC, and EAEC pathotypes but not in EHEC [6,53]. Thus, it is not surprising that the clinical isolates of diarrheal origins would not possess these two genes. Isolates AW1.7, 53, and 63 lacking the *papC* and *Agn43* genes may be attributed to the vastly different ecological niches that they circulate in compared to UPEC, SEPEC, and EAEC strains. Biofilm forming UPEC and SEPEC strains are commonly found in catheters and other medical devices [54,55] whereas EAEC strains have been identified in contaminated food and drinking water [56] but not in slaughterhouses and wastewater [57]. Interestingly, members of the *fim* operon, *fimA*, *fimB*, and *fimE* were detected in isolates 53, 63, 111, and 128 but not in AW1.7 or 8354. Type 1 fimbriae are proteinaceous filamentous adhesins that are important for attachment to abiotic surfaces [58]. Its prevalence has been reported to be high in UPEC, EPEC, and SEPEC pathotypes but similarly has not been investigated thoroughly in EHEC [6]. It is possible that type 1 fimbriae may not play a significant role in biofilm formation in EHEC pathotypes, as reflected in the data presented in this study. The last biofilm formation-associated genes of interest were those comprising the *mrk* operon. Encoding for type 3 fimbriae, the operon was identified as an additional member in the LHR in a heat resistant *E. coli* strain isolated from Swiss raw milk cheese [43]. The *mrk* operon is commonly present in *Klebsiella pneumoniae* but rarely reported in *E. coli*. The lack of the *mrk* operon suggests that the LHR present in the heat resistant isolates likely originates from a different source than the one identified by Marti et al. [43,59]. A limitation of this study is the genetic determination of biofilm formation-associated genes without corresponding expression detection; future research on characterizing expression of these genes will allow us to gain a deeper insight into biofilm formation in *E. coli* pathotypes that have yet to be extensively studied.

## 5. Conclusions

In this study, we were able to confirm biofilm formation in heat resistant *E. coli* isolates from environmental and clinical sources in Alberta using the two-component apparatus and demonstrate that the LHR likely does not contribute to increased biofilm forming potential. The findings obtained in this study provide further evidence that biofilms are not regarded as an essential virulence factor for EHEC survival and pathogenesis [60]. Weaker biofilm forming potential of clinical *E. coli* strains compared to environmental *E. coli* strains is likely due to higher adaptation to various stress conditions the latter encounter in their ecological niches. The identification of pathogenic *E. coli* that possess the LHR and tolerance to multiple environmental stressors illustrates the threat they pose on food safety at various points in the farm-to-fork continuum. Further study on the contribution of heat resistant *E. coli* in human foodborne infection may potentially identify new sources of contamination and shortcomings in current pathogen inactivation methods used in the food processing industry.

## Figures and Tables

**Table 1:** Biofilm formation-associated genes.

Gene	Gene ID	Description	References
*csgB*	947391	Minor curlin subunit	[18]
*csgA*	949055	Major curlin subunit	[19]
*csgC*	945623	Inhibitor of *CsgA* amyloid formation	[20]
*csgD*	949119	DNA-binding transcriptional dual regulator of genes involved in curli assembly, transport, and structure components	[19,21]
*csgE*	945711	Specificity factor in the *CsgG* mediated outer membrane translocation of the curli subunits	[22]
*csgF*	945622	Acts in conjunction with *CsgB* to initiate curli subunit polymerisation	[22]
*csgG*	945619	Forms the secretion channel for curli subunits, providing stability to *CsgA* and *CsgB* during assembly	[19]
*hha*	945098	Represses the transcription of fimbrial genes	[23]
*bcsA*	948053	Catalytic subunit of cellulose synthase	[24]
*bcsB*	948045	Cellulose synthase periplasmic subunit	[24]
*pgaC*	945606	Poly-N-acetyl-D-glucosamine (PGA) synthase subunit mediating translocation and/or docking of PGA to the cell surface	[25]
*papC*	7152342	Export and assembly of pili subunits across the outer membrane needed for formation of P fimbriae	[26]
*agn43*	946540	Autoaggregation factor promoting adhesion	[27,28]
*fimA*	948838	Major subunit of type 1 fimbriae	[29]
*fimB*	948832	Recombinase regulating type 1 fimbriae production	[30]
*fimE*	948836	Recombinase regulating type 1 fimbriae production	[30]
*mrkA*	8569608	Major subunit of plasmid encoded type 3 fimbriae	[31,32]
*mrkB*	8569607	Type 3 fimbriae chaperone involved in assembly and anchorage of fimbrial filaments	[32,33]
*mrkC*	8569606	Type 3 fimbriae usher involved in assembly and anchorage of fimbrial filaments	[32,33]
*mrkD*	8569605	Adhesin subunit of type 3 fimbriae	[31,32]
*mrkF*	8569604	Stabilizes intact type 3 fimbriae	[31,32]

**Table 2:** Multivariate linear regression analysis of factors associated with biofilm formation in *Escherichia coli* isolates.

Variable	Effect Size (95% CI) Stratified by Isolate			
	AW1.7	53	63	111
Bacterial inoculum	0.021 ^†^ (0.007, 0.035)	−0.011 * (−0.020, −0.003)	0.001 (−0.006, 0.007)	0.008 * (0.001, 0.015)
Temperature (37 °C)	−0.053 (−0.201, 0.096)	−0.050 (−0.144, 0.045)	0.040 (−0.028, 0.108)	−0.194 ^‡^ (−0.232, −0.157)
LB broth concentration	−0.006 ^‡^ (−0.007, −0.004)	0.002 ^‡^ (0.001, 0.004)	0.005 ^‡^ (0.004, 0.005)	0.002 ^‡^ (0.001, 0.002)
Temperature:LB broth concentration	0.016 ^‡^ (0.013, 0.018)	0.002 * (0.000, 0.004)	−0.002 ^‡^ (−0.003, −0.001)	-

Reference temperature of 24 °C. * Significant at *p* < 0.05, ^†^ significant at *p* < 0.01, ^‡^ significant at *p* < 0.001; dash, not applicable.

**Table 3:** Genetic determination of biofilm formation-associated genes in heat resistant *Escherichia coli.*

Isolate	Gene *								
	*hha*	*bcsA*	*bcsB*	*pgaC*	*papC*	*Agn43*	*fimA*	*fimB*	*fimE*
AW1.7	+	+	+	+	-	-	-	-	-
53	+	+	+	+	-	-	+	+	+
63	+	+	+	+	-	-	+	+	+
111	+	+	+	+	-	-	+	+	+
128	+	-	-	+	-	-	+	+	+
8354	+	+	+	+	-	-	-	-	-

* All genes comprising the *csgBAC* and *csgDEFG* operons were present in the isolates. All genes comprising the *mrkABCDF* operon were absent in the isolates.
